# Variable Relative Biological Effectiveness of Protons in the Rat Spinal Cord: Measurements and Comparison With Model Calculations

**DOI:** 10.1016/j.adro.2025.101809

**Published:** 2025-05-16

**Authors:** Christin Glowa, Maria Saager, Lisa Hintz, Rosemarie Euler-Lange, Peter Peschke, Stephan Brons, Kaya Hilt, Thomas Friedrich, Michael Scholz, Hans Liew, Andrea Mairani, Christian P. Karger

**Affiliations:** a Department of Medical Physics in Radiation Oncology, German Cancer Research Center (DKFZ), Heidelberg, Germany; b National Center for Radiation Research in Oncology (NCRO), Heidelberg Institute for Radiation Oncology (HIRO), Heidelberg, Germany; c Department of Radiation Oncology and Radiotherapy, University Hospital Heidelberg, Heidelberg, Germany; d Faculty of Biosciences, Heidelberg University, Heidelberg, Germany; e Department of Radiooncology/Radiobiology, German Cancer Research Center (DKFZ), Heidelberg, Germany; f Heidelberg Ion Beam Therapy Center (HIT), Heidelberg, Germany; g Department of Biophysics, Helmholtz Center for Heavy Ion Research (GSI), Darmstadt, Germany; h Clinical Cooperation Unit Translational Radiation Oncology, German Cancer Consortium (DKTK) Core-Center Heidelberg, National Center for Tumor Diseases (NCT), Heidelberg University Hospital (UKHD), German Cancer Research Center (DKFZ), Heidelberg, Germany; i Division of Molecular and Translational Radiation Oncology, Heidelberg Faculty of Medicine (MFHD), Heidelberg University Hospital (UKHD), Heidelberg Ion Beam Therapy Center (HIT), Heidelberg, Germany; j National Centre of Oncological Hadrontherapy (CNAO), Medical Physics, Pavia, Italy

## Abstract

**Purpose:**

To determine the relative biological effectiveness (RBE) in the rat spinal cord after 6 fractions of protons as a function of linear energy transfer (LET) and dose.

**Methods and Materials:**

The rat spinal cord was irradiated at 4 different positions of a 6 cm spread-out Bragg peak using 6 fractions of protons (LET: 1.4, 2.7, 3.9, and 5.5 keV/µm). Dose-response curves were established for the endpoint paresis grade 2, and the RBE was calculated based on the dose at 50% effect probability. Including data with single and split doses, the measured RBE values were compared with predictions from 4 mechanistic, 3 phenomenological, and 2 patient-derived variable RBE models.

**Results:**

With increasing LET, the dose at 50% effect probability decreased from 51.3 Gy to 43.3 Gy, resulting in a rise in the RBE from 1.11 to 1.32. The biologically equivalent dose decreased markedly between the 2 proximal and 2 distal spinal cord positions, resulting in extrapolated maximum RBE values of up to 1.87 in the limit of zero dose per fraction. The α/β values ranged between 1.5 Gy and 4.2 Gy. At 3.9 and 5.5 keV/µm, the RBE increased with decreasing dose, and at 1.8 Gy per fraction, the RBE was extrapolated to 1.40 and 1.42, respectively. The agreement between predicted and measured RBE varied between the different models.

**Conclusions:**

A fixed RBE of 1.1 provides a good approximation up to the center of the spread-out Bragg peak; however, at 3 mm from the distal end, the RBE increases markedly and may reach values above 1.4 at clinical fraction schedules. Using predictions from a variable RBE model may, therefore, be reasonable; however, the model and model parameters should be carefully selected, ideally as a consensus among the proton therapy centers.

## Introduction

Proton therapy is an established radiation modality for the treatment of tumors localized close to sensitive, normal tissue structures.^1^^,^^2^ The favorable depth dose distribution (spread-out Bragg peak [SOBP]), together with state-of-the-art pencil beam scanning, allows for delivering highly conformal dose distributions.^3^ Since the first clinical application in 1954, more than 350,000 patients have been treated with protons, and more than 110 proton therapy centers worldwide are currently in operation.^4^

Protons exhibit an increased relative biological effectiveness (RBE) with respect to photons, and currently, a fixed RBE of 1.1 is used at all centers, independent of linear energy transfer (LET), dose per fraction, and other factors.^5^ While this value is considered to be a conservative average for tumor control,^5^ experimental data indicate that the RBE increases to significantly higher and dose-dependent values at the end of the SOBP.^6^ This raised a discussion about the necessity of using variable RBE models or revised treatment planning strategies.^7^, ^8^, ^9^ Different RBE models have been developed^10^, ^11^, ^12^, ^13^, ^14^, ^15^, ^16^, ^17^, ^18^, ^19^, ^20^, ^21^, ^22^; however, prior to their clinical application in proton therapy, evidence for a variable RBE needs to be established.^23^ The strongest evidence for a variable RBE originates from clonogenic cell survival studies providing uniform LET conditions in the absence of volume- or other tissue-related effects; however, the clinical relevance of these in vitro findings remains less clear.^5^^,^^6^ Although several clinical studies have been performed,^5^ it is difficult to separate the impact of LET, dose, and other risk factors on the radiation response and to derive accurate RBE values, especially for the late-responding central nervous system (CNS).^24^, ^25^, ^26^

To study the late radiation response of the CNS, the spinal cord of primates, pigs, rats, and mice has been used as animal models.^27^, ^28^, ^29^, ^30^ In addition, early and late skin effects have been quantified in mice.^31^, ^32^, ^33^ The rat spinal cord has been used to systematically investigate the RBE of different ion types as a function of LET and dose.^34^, ^35^, ^36^, ^37^ In a previous study,^34^ the RBE of single and split proton doses was determined at 4 positions of the rat spinal cord within a 6 cm SOBP. As a result, an increase in the RBE from the plateau to the distal edge of the SOBP of approximately 0.15 was found; however, a consistent dose-dependence could not be resolved. To study the RBE at lower fraction doses, the same setup was used to determine the RBE in the rat spinal cord after 6 fractions of protons. Including the data for single and split doses, LET- and dose-dependence were analyzed and compared using predictions of 4 mechanistic, 3 phenomenological, and 2 patient-derived variable RBE models.

## Methods and Materials

### Animals

In total, 135 young adult female Sprague-Dawley rats (Charles River, Sulzfeld, Germany) at a mean age of 10 (range ± 2) weeks and a mean ± SD weight of 228 ± 20 g were used for this study, including 5 unirradiated controls. A gaseous mixture of 4% Sevoflurane (Abbott, Wiesbaden, Germany) and oxygen at 2 L/min was used to keep animals under anesthesia during irradiation. The rats were housed under standard conditions at the Center for Preclinical Research of the German Cancer Research Center, and all experiments were approved by the governmental review committee on animal care (reference number 35-9185.81/G-272/19).

### Experimental setup

The setup of the spinal cord irradiation with 6 fractions of protons was the same as previously used for single and split proton doses,^34^ and only a brief description will be given here. Animals were irradiated in a hanging position with a horizontal beam coming from the ventral direction.^38^^,^^39^ Animals were positioned under video control by means of room lasers and marks on the fixation device using a remotely controlled conveyor belt. The segments C1 to C6 of the rat cervical spinal cord were irradiated with protons at 4 different positions (35, 100, 120, and 127 mm) of a 6 cm SOBP ranging from 70 to 130 mm water-equivalent depth.^34^ The corresponding dose-averaged LET values were 1.4, 2.7, 3.9, and 5.5 keV/µm, respectively. The range of the ions was adjusted with appropriate polymethyl-methacrylate boli. Protons were delivered using the intensity-controlled raster scanning technique^40^ at the Heidelberg Ion Beam Therapy Center (HIT).^41^ Treatment fields were optimized with the treatment planning system (TPS) TRiP (Treatment planning for particles) in terms of absorbed dose,^42^ including multiple Coulomb scattering. The field size (6 × 11 mm^2^ at the 90% isodose) was the same as in our previous study with single and split proton doses.^34^ Specified irradiation doses refer to the maximum dose at the field center at the respective spinal cord position. Absolute dose measurements with a pinpoint ionization chamber (TM31009, PTW Freiburg, Germany) were used to normalize the treatment plan to the prescribed dose at the respective depth.^34^

### Proton irradiations

At each position within the SOBP, the spinal cord was irradiated with 6 fractions of protons delivered on consecutive days. The total absorbed doses are displayed in Table 1. Irradiations were performed with increasing dose levels using 5 animals per dose group. Doses were selected to cover 0% to 100% complication probabilities. Five animals served as sham-treated controls.

**Table 1:** Total absorbed dose and number of animals used for the experiments. Each dose level contained 5 animals, and 5 sham-treated animals served as controls

Spinal cord position within SOBP (mm)	LET (keV/µm)	Dose levels (Gy)	Total no. of animals
6 Fractions			
35	1.4	48*, 49.5†, 51, 52.5, 54, 55.5	30
100	2.7	43, 44.5, 46, 47.5, 49, 50.5†, 52, 53.5	40
120	3.9	42.5, 44‡, 45.5, 47, 48.5†, 50	30
127	5.5	40, 41.5‡, 43*, 44.5, 46, 47.5†	30
Controls	-	-	5

*Abbreviations:* LET = linear energy transfer; SOBP = spread-out Bragg peak.

Animals had to be excluded because of the development of *spinal tumor (226 d and 234 d)

† mammary carcinoma (161 d, 211 d, 245 d, and 211 d), and ‡lipoma (228 d and 272 d).

### Follow-up and biological endpoint

After irradiation, rats were checked for weight and general condition once a week. The biological endpoint was paresis grade 2, meaning that both forelimbs show signs of paralysis within 300 days.^39^ Rats developing this endpoint were euthanized and scored as responders.

### Data analysis

The logistic dose-response model describing the probability of the selected endpoint at the dose D

(1) $$P\left(D\right)=\frac{e^{b_{0}+b_{1}D}}{1+e^{b_{0}+b_{1}D}}$$

was adjusted to the actuarial proton response rates to determine the parameters $b_{0}$ and $b_{1}$, from which the effective dose at 50% effect probability, ED_50_, can be calculated as

(2) $$ED_{50}=-\frac{b_{0}}{b_{1}}.$$

This was performed at each spinal cord position in the SOBP. RBE values were then determined as the ratio of the ED_50_ value obtained from a previous 6-fraction reference experiment with photons^37^ and those of the present proton experiment.

In addition, the biologically effective dose at 50% effect probability (BED_50_) and the parameter α/β of the linear-quadratic model^43^ were simultaneously determined by adjusting the generalized logistic model

(3) $$P\left(D\right)=\frac{e^{b_{0}+b_{1}D+b_{2}Dd}}{1+e^{b_{0}+b_{1}D+b_{2}Dd}}=\frac{e^{b_{0}+b_{1}D\left(1+\frac{d}{b_{1}/b_{2}}\right)}}{1+e^{b_{0}+b_{1}D\left(1+\frac{d}{b_{1}/b_{2}}\right)}}=\frac{e^{b_{0}+b_{1}BED}}{1+e^{b_{0}+b_{1}BED}},$$

to the actuarial proton response rates for 1, 2, and 6 fractions using additional data for single and split doses from a previous study.^34^ BED_50_ and α/β could then be calculated from the fit parameters $b_{0}$, $b_{1}$, and $b_{2}\;$by

(4) $$BED_{50}=-\frac{b_{0}}{b_{1}}\;\text{and}\;\frac{\alpha }{\beta }=\frac{b_{1}}{b_{2}}.$$

From the ratio of BED_50_ values for photons and protons, the upper limit of the RBE (RBE_max_) in the limit of zero fraction dose was estimated. This same method has been previously applied to photon data.^36^^,^^37^ The dependence of the experimentally obtained RBE values on dose per fraction was inter- and extrapolated using the fitted BED_50_ and α/β values of photons and protons to calculate the isoeffective doses at a given fraction dose. The RBE at this fraction dose was then calculated as the ratio of the isoeffective doses for photons and protons.

### RBE calculation

For comparison with the measured data, the RBE was calculated at the 4 spinal cord positions within the SOBP using the local effect model (LEM I, clinically applied for carbon ions),^10^ the more recent version LEM IV (employing the “full simulation approach”),^11^^,^^12^ the modified microdosimetric kinetic model (mMKM, clinically applied for carbon ions^13^^,^^14^ and recently also for helium ions^15^), and the UNIVERSE (UNIfied and VERSatile bio Response Engine) model.^16^^,^^17^ In addition, the 3 phenomenological models from Carabe (CRB) et al^18^, Wedenberg (WDB) et al ^19^, and McNamara (MNM) et al^20^, as well as 2 patient-derived models from Bahn et al^25^ and Eulitz et al,^26^ were employed. The RBE was calculated at dose levels given by the ED_50_ values for protons estimated in the present and a previous^34^ study. Calculations were performed with standard parameters for LEM I (α/β = 2 Gy, α = 0.1 Gy^−1^, transition dose D_t_ = 30 Gy), LEM IV (α/β = 2 Gy, α = 0.003 Gy^−1^, D_t_ = 22 Gy),^44^ and mMKM (α/β = 2 Gy, α = 0.003 Gy^−1^, domain radius R_d_ = 0.3 µm, radius of cell nucleus R_n_ = 3.6 µm).^45^^,^^46^ For UNIVERSE, the lethality parameters were set to K_i_ = 0.0 and K_c_ = 0.0132 (probabilities that an isolated or complex DNA double strand break inactivates a cell), while all other parameters were kept at their standard setting.^16^^,^^17^ Compared with a previous study,^17^ K_i_ and K_c_ were changed to fix α/β = 2 Gy for photons, and as a result, α = 0.003 Gy^-1^ was obtained. The parameters of the phenomenological^18^, ^19^, ^20^ and patient-derived models^25^^,^^26^ were taken from the respective references. The α/β value was set to 2 Gy for the phenomenological models, while α/β was not an explicit parameter in the patient-derived models.

### Statistics

The logistic model was adjusted using the maximum likelihood fitting procedure in STATISTICA.^47^ Incomplete follow-up of animals was considered in the maximum likelihood fit using the method of effective sample sizes,^48^ which corrects the number of treated and responding animals to match actuarial response rates and their variances. SEs of ED_50_, RBE, and RBE relative to plateau, BED_50_, RBE_max_, and α/β were calculated by error propagation, considering the correlation of the fit parameters of the logistic model in the case of ED_50_ and BED_50_. In addition, 90% confidence limits were calculated using Fieller’s Theorem.^49^ Testing of the slopes of the linear regressions for significance used a *P* value of .05.

## Results

Irradiations were well tolerated by the animals. Some animals had to be excluded from the experiments because of the spontaneous development of mammary carcinoma or for other reasons (Table 1). The latency time until the onset of endpoint paresis grade 2 is shown in Fig. E1. Based on the slope of the linear regression, no significant dependence on LET was found for the minimum (*P* = .75) and mean (*P* = .19) latency times.

**Figure 1:**
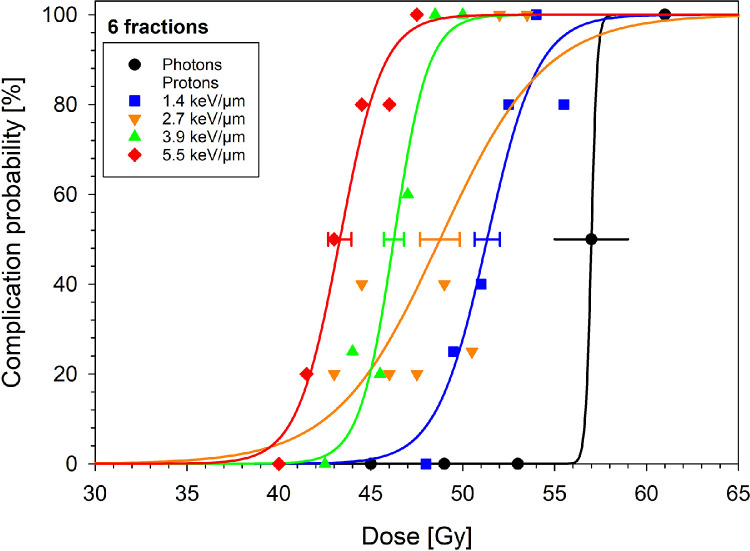
Dose-response curves for the endpoint paresis grade 2 after irradiating the rat spinal cord at 4 different positions within the spread-out Bragg peak with 6 fractions of protons. Error bars indicate the SE of the effective dose at 50% effect probability. The dose-response curve for photons was taken from Karger et al.^37^

**Figure 2:**
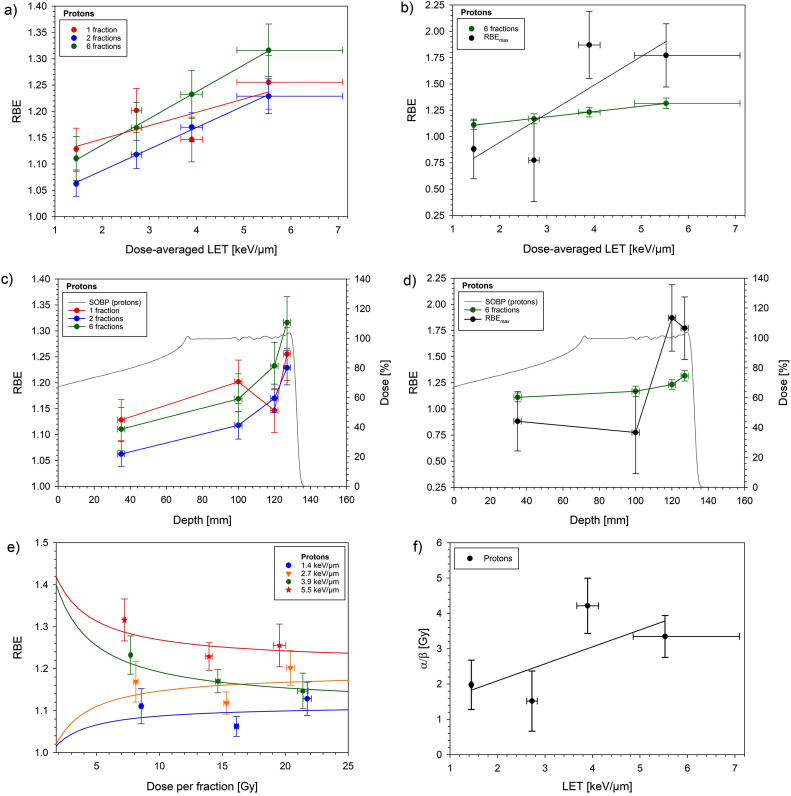
Relative biological effectiveness (RBE) of the rat spinal cord after proton irradiation as a function of linear energy transfer (LET) (a, b), water-equivalent depth (c, d), and dose per fraction (e), as well as the LET dependence of α/β (f). LET and depth dependence of the RBE is displayed for 6 fractions in comparison with the previously measured proton data for single and split doses.^34^ LET dependence of the RBE and α/β was fitted by linear regression. The dependence of the RBE on dose was inter- and extrapolated using the fitted biologically effective dose at 50% effect probability (BED_50_) and α/β values of protons and photons to calculate the isoeffective doses and RBE at a given fraction dose (see text). The upper limit of the RBE (RBE_max_) values was obtained as the ratio of the BED_50_ values of photons and protons. Horizontal error bars display the LET uncertainty (a, b, f) based on a 2 mm range uncertainty (c, d) or the SE of the effective dose at 50% effect probability per fraction (e), while the vertical error bars indicate the SE of the RBE (a-e) or α/β (f). *Abbreviation:* SOBP = spread-out Bragg peak.

The measured dose-response curves for protons are shown in Fig. 1 in comparison with the photon reference curves. With increasing LET, the curves were shifted to lower doses. Figure 2 displays the measured RBE as a function of LET (Fig. 2a, b), water-equivalent depth (Fig. 2c, d), and dose per fraction (Fig. 2e), as well as the LET dependence of the measured α/β value (Fig. 2f). For 6 fractions of protons, the ED_50_ decreased from 51.3 Gy at 1.4 keV/µm to 43.3 Gy at 5.5 keV/µm, leading to a rise in the RBE from 1.11 to 1.32 (Table 2, ED_50_). Relative to the plateau, the RBE at 2.7, 3.9, and 5.5 keV/µm increased by a factor (± SE, 90% CL) of 1.05 ± 0.03 (1.01-1.10), 1.11 ± 0.02 (1.08-1.14), and 1.19 ± 0.02 (1.15-1.22), respectively. BED_50_ showed a marked decrease between the 2 proximal and 2 distal spinal cord positions, resulting in a strong increase in the extrapolated RBE_max_ of up to 1.87 (Table 2, BED_50_). Estimated α/β values ranged between 1.5 Gy and 4.2 Gy between the 2 proximal and 2 distal spinal cord positions (Table 2, α/β). At LET values of 3.9 and 5.5 keV/µm, the RBE increased with decreasing doses, and at doses of 1.8 Gy per fraction, the estimated RBE reached values of 1.40 and 1.42, respectively (Fig. 2e). No indication of an increase in RBE with decreasing dose was found for the 2 lowest LET values.

**Table 2:** The effective dose at 50% effect probability and relative biological effectiveness values for 6 fractions derived from data in this study, as well as the biologically effective dose at 50% effect probability, the upper limit of the relative biological effectiveness, and α/β values derived from data in this and a previous study^37^ that employed single and split doses

Study	Measured parameters	
*6 Fx*	*ED_50_ ± SE (90% CL) [Gy]*	*RBE ± SE (90% CL)*
Photons*	57.0 ± 2.0 (-)	-
Protons		
1.4 keV/µm	51.3 ± 0.7 (49.7-52.6)	1.11 ± 0.04 (1.04-1.18)
2.7 keV/µm	48.8 ± 1.1 (45.8-54.0)	1.17 ± 0.05 (1.09-1.25)
3.9 keV/µm	46.2 ± 0.5 (45.1-47.4)	1.23 ± 0.05 (1.16-1.31)
5.5 keV/µm	43.3 ± 0.6 (42.0-44.5)	1.32 ± 0.05 (1.23-1.40)
*1, 2, and 6 Fx*	*BED_50_ ± SE (90% CL) [Gy]*	*RBE_max_ ± SE (90% CL)*
Photons†	244.9 ± 24.3 (208.2-293.3)	-
Protons		
1.4 keV/µm	277.6 ± 84.9 (182.9-633.1)	0.88 ± 0.28 (0.56-1.79)
2.7 keV/µm	316.0 ± 157.6 (175.7-4941.8)	0.77 ± 0.39 (0.41-4.27)
3.9 keV/µm	130.9 ± 18.1 (106.4-175.0)	1.87 ± 0.32 (1.43-2.52)
5.5 keV/µm	138.2 ± 19.0 (111.8-184.1)	1.77 ± 0.30 (1.35-2.38)
*1, 2, and 6 Fx*	α/β *± SE (90% CL) [Gy]*	
Photons†	2.8 ± 0.4 (2.2-3.5)	
Protons		
1.4 keV/µm	2.0 ± 0.7 (0.8-3.3)	
2.7 keV/µm	1.5 ± 0.9 (0.1-3.0)	
3.9 keV/µm	4.2 ± 0.8 (2.9-5.6)	
5.5 keV/µm	3.3 ± 0.6 (2.3-4.4)	

Values include SEs and 90% CLs.

*Abbreviations:* BED_50_ = biologically effective dose at 50% effect probability; CL = confidence limit; ED_50_ = effective dose at 50% effect probability; Fx = fraction; RBE = relative biological effectiveness; RBE_max_ = upper limit of the relative biological effectiveness.

⁎ Data from.^37^

† Derived in ^36^ based on data from.^37^

**Figure 3:**
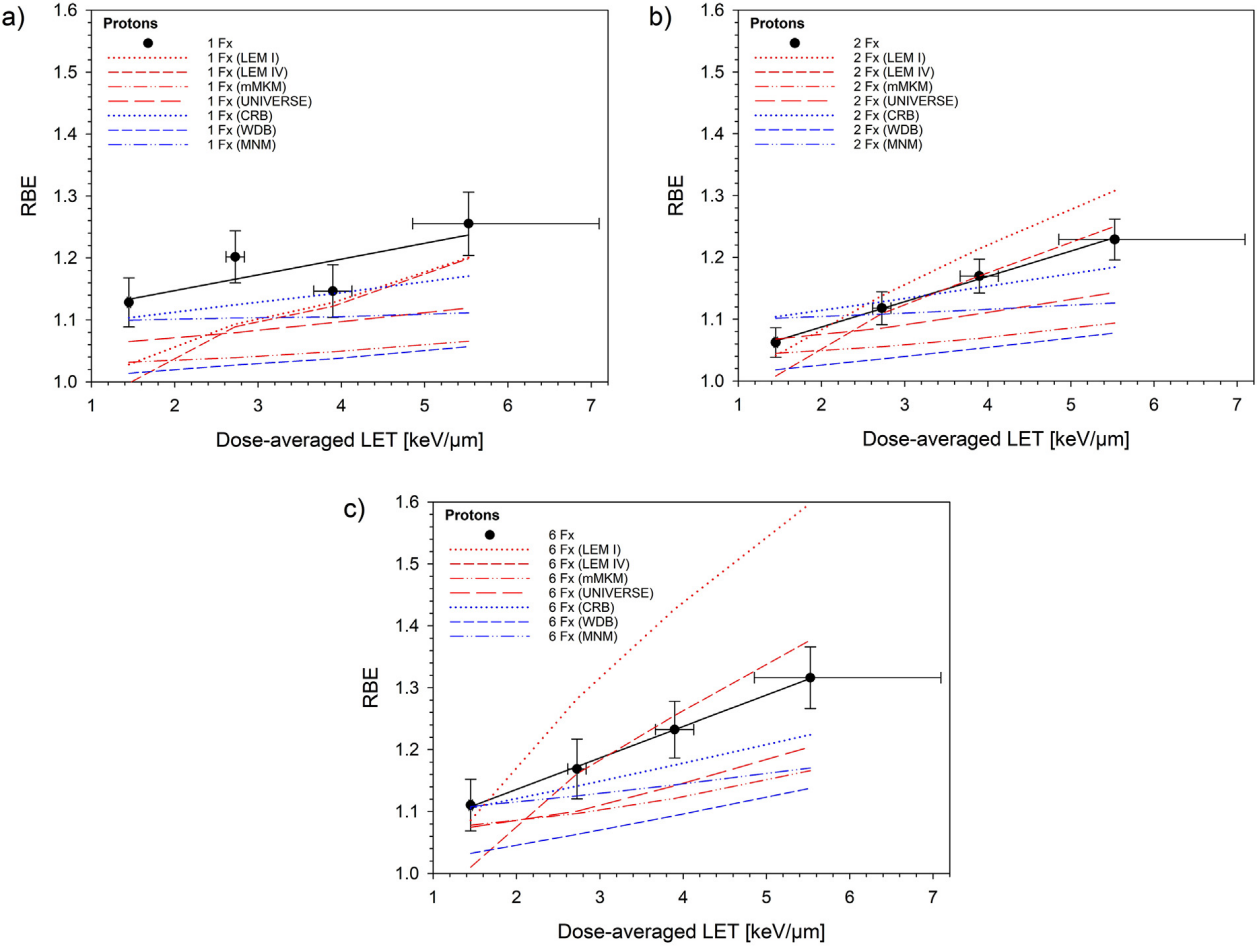
Comparison of model-predicted and measured relative biological effectiveness (RBE) of protons as a function of linear energy transfer (LET) for single (a), split (b), and 6 (c) fractions (Fx). Four mechanistic (local effect model [LEM] I, LEM IV, modified microdosimetric kinetic model [mMKM], and UNIVERSE) and 3 phenomenological (CRB, WDB, MNM) models were used. Experimental data are the same as in Fig. 2a. *Abbreviation:* CRB = Carabe model; MNM = McNamara model; UNIVERSE = UNIfied and VERSatile bio Response Engine; WDB = Wedenberg model.

**Figure 4:**
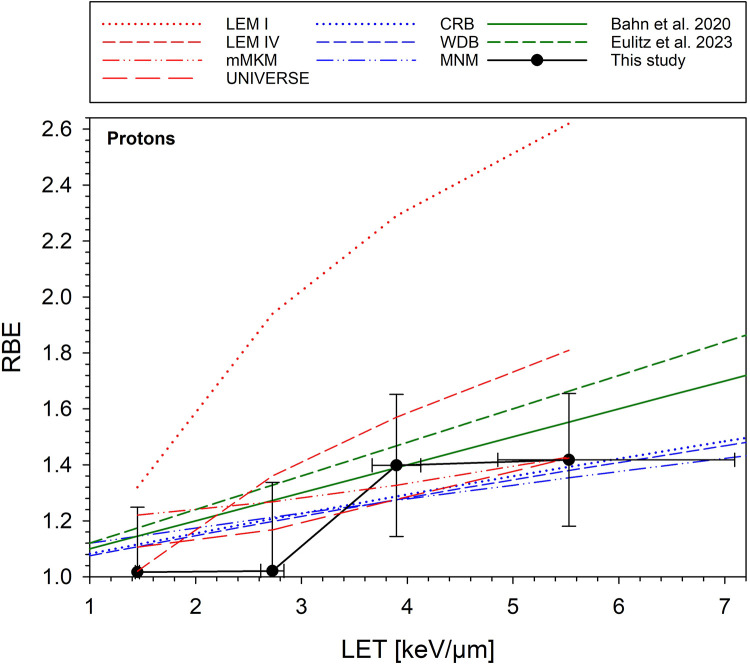
Relative biological effectiveness (RBE) of protons extrapolated from experimental data to clinically applied doses of 1.8 Gy per fraction (corresponding to 2 Gy [RBE] using RBE = 1.1) compared with predictions of 4 mechanistic (local effect model [LEM] I, LEM IV, modified microdosimetric kinetic model [mMKM], and UNIVERSE), 3 phenomenological (CRB, WDB, and MNM), and 2 additional patient-derived (Bahn et al^25^ and Eulitz et al^26^) models. Horizontal error bars display the linear energy transfer (LET) uncertainty based on a 2 mm range uncertainty. The error bars for the RBE were determined by linearly interpolating the SEs of the upper limit of the RBE (RBE_max_) and RBE for 6 fractions (Table 2) as a function of dose per fraction. *Abbreviation:* CRB = Carabe model; MNM = McNamara model; UNIVERSE = UNIfied and VERSatile bio Response Engine; WDB = Wedenberg model.

Figure 3 compares the predicted and measured RBE as a function of LET for the 4 mechanistic and 3 phenomenological models at 1, 2, and 6 fractions. A comparison of predicted and measured RBE as a function of depth and fraction dose is shown in Fig. E2 (RBE vs depth) and Fig. E3 (RBE vs dose), respectively. Figure E4 displays the deviations per model for the 4 LET values at 1, 2, and 6 fractions. Numerical values of the deviations between predicted and measured RBE, averaged over all fractionation schedules, are displayed separately for the SOBP and plateau positions in Table E1. The table additionally shows the respective SD, which is a measure of the uniformity of the deviations. The smallest mean deviations were obtained for LEM IV and the CRB model (SOBP) and the MNM and CRB models (plateau). The highest uniformity of the deviations was found for the UNIVERSE and CRB models (SOBP) and the CRB and MNM models (plateau). A comparison of predicted and experimental RBE extrapolated to clinically applied doses of 1.8 Gy (corresponding to 2 Gy [RBE] with RBE = 1.1) is displayed in Fig. 4 for the 4 mechanistic, 3 phenomenological, and 2 additional patient-derived models.

## Discussion

An end-of-range increase in proton RBE has been clearly demonstrated in several cell lines, and values well above 1.5 have been found.^6^^,^^50^ These high values, however, were obtained in homogeneous media at residual ranges below 100 µm, resulting in high LET values, which would be significantly lower in heterogeneous tissue because of the superposition of protons of different ranges. Nevertheless, increased LET and RBE values may also be expected in patients, especially in the safety margin behind the tumor, if normal tissue (eg, of the CNS) with low α/β values is contained. While there are some indications that toxicity in patients is caused by an LET-related increase in RBE, it remains difficult to separate the effects clearly from LET, dose, volume, and other risk factors like irradiation of periventricular regions.^25^^,^^26^

The rat spinal cord is a well-established animal model to measure the radiation response of the CNS to the local beam quality, and by comparing the response of photons and ions, the RBE can be quantified. To determine the RBE for the rat spinal cord without bias, the length of the irradiated segment was selected to be larger than 8 mm since the volume effect can then be neglected in rats.^51^^,^^52^ Previous experiments for single and split doses already indicated a moderate increase in the RBE by approximately 0.15 from the plateau to the distal SOBP position, and in the present 6-fraction study, the RBE at the distal positions increased further to 1.32 while remaining nearly constant in the plateau and mid-SOBP positions.

A few other studies quantified the response of normal tissue to proton irradiations^30^, ^31^, ^32^; however, only 2 compared ED_50_ for different SOBP positions, and only 1 derived RBE values by including a photon experiment. Overgaard et al^32^ measured single-dose proton RBE values at mid- and distal SOBP positions of 1.06 and 1.15 for acute skin effects in mice, respectively, and 1.16 and 1.26 for late skin effects, respectively. Denbeigh et al^30^ measured proton ED_50_ ratios between the entrance of a mono-energetic Bragg peak and the center of a 4 mm SOBP in the mouse spinal cord and reported values of 1.10 for single and 1.19 for 18 fractions. Assuming an RBE of 1.10 at the entrance, this corresponds to RBE values in the 4 mm SOBP of 1.21 and 1.31 at single and 18 fractions, respectively. These values are comparable with the distal RBE values obtained in this and a previous study^34^ in the rat spinal cord, although at different fraction doses. Overall, the fraction dose of all these experiments is still rather high. Although associated with additional uncertainty, we, therefore, extrapolated our RBE data to lower fraction doses and obtained 1.87 as the upper limit (RBE_max_) and 1.42 at 1.8 Gy per fraction at the distal position, where the residual proton range is about 3 mm. Therefore, the RBE may be even larger for residual ranges below 3 mm. On the other hand, the clinically applied value of 1.1 was confirmed as a good approximation in the plateau and at the mid-SOBP.

Unexpectedly, the RBE in our previous study^34^ was about 0.07 higher for the single than the split dose experiment in the plateau and mid-SOBP, while it was comparable at the distal SOBP positions. This is most likely because of possible small experimental uncertainties (eg, animal positioning or beam settings), which limit the possibility of resolving such small differences. The 2 unexpectedly higher single-dose RBE values also affected the extrapolation of the RBE toward small fraction doses in the plateau and mid-SOBP positions. With respect to this, the decrease in the RBE at small doses, as predicted by the extrapolation with the linear-quadratic model, should therefore be considered rather as an indication of dose-independence, while the increase at the 2 distal positions is more reliable because of the clearly visible increase in the measured RBE at 6 fractions.

This study, as well as a previous proton experiment,^34^ used historical photon data^37^^,^^39^ as a reference to determine the RBE. This is justified because the values of ED_50_ for single photon and carbon ion doses have been shown to remain unchanged within 0.3 Gy after 16 years and within 0.1 Gy after 3 years, respectively.^53^ In addition, the RBE values obtained for protons can be directly set in relation to the RBE of other ions, where the same photon reference data have been used.^35^, ^36^, ^37^^,^^39^ All these experiments used the same experimental setup and the same fixation device. In addition, the age and weight of the animals varied only within narrow ranges, and from a previous study, it is known that ED_50_ is not affected by age if the animals are older than 3 weeks.^54^ However, some limitations of our study should be noted: (1) the guidance of the increase in the 6-fraction photon dose-response curve by only 3 data points, (2) an apparently increased heterogeneity in the proton response at 2.7 keV/µm, leading to a more shallow dose-response curve, but still to a smooth RBE vs LET dependence, and (3) the use of rather low sample sizes per dose group as a trade-off between experimental uncertainty and logistic effort. While (1) is a result of a previous study,^37^ no specific reason for (2) could be found. All experiments were performed under the same conditions, following the same experimental protocols. Furthermore, the parameter ED_50_ of sigmoid dose-response models is rather robust against changes in the slope. Overall, the uncertainties introduced by (1) and (2), as well as by the sample size in general in (3), are reflected by the SE of ED_50_; when propagating this uncertainty, the SE of the RBE still remained ≤0.05, and for the relative increase in the proton RBE with depth, the SE was even reduced by a factor of 2. This uncertainty is sufficiently small to discriminate clinically relevant changes of the RBE with increasing LET.

Although the uncertainty is quite large, a moderate increase in α/β between the 2 proximal and 2 distal SOBP positions was found in the present study. While the α/β values in the plateau and mid-SOBP positions agree well with published data for photons,^5^^,^^37^ the increase in α/β toward the distal end of the SOBP indicates that repair of radiation damage may be suppressed to some extent, but to a lesser extent than for high LET ions.^36^

Overall, there is no doubt about the end-of-range increase in the RBE from experimental studies, and most likely, the RBE will increase to values well above 1.1 but will also stay below the maximum values measured in cell studies.^6^^,^^50^ The experimentally obtained RBE values were used to benchmark selected mechanistic,^10^, ^11^, ^12^^,^^16^^,^^17^^,^^46^^,^^55^ phenomenological,^18^, ^19^, ^20^ and patient-derived^25^^,^^26^ variable RBE models that could be used to account for the variable RBE. This list of tested models, however, could be further extended to other new or updated models.^21^^,^^22^ The present and a previous^34^ study provide the experimental database for this. As can be seen in Fig. 3 and Fig. E4, LEM IV most accurately predicts the RBE in the SOBP region among the mechanistic models, whereas, for the phenomenological models, predictions of the CRB model are closest to the experimental data. However, there are small to moderate systematic negative deviations for most models and/or systematic trends with increasing LET for each fractionation schedule. Only in one case (LEM I) was there also a strong trend as a function of fractionation. When selecting a model for clinical application, small and uniform deviations in the SOBP and preferably in the plateau are desired. Notably, the CRB model meets both criteria; however, other models also exhibit comparable agreements, and several models could, therefore, be used in patients. It is noteworthy that at the high doses applied in this study, dose-rate effects within the SOBP may become additionally relevant. Their consideration could improve the accuracy of RBE models, as shown in a previous study.^56^

Although associated with additional uncertainty, the measured RBE values were extrapolated to lower doses, allowing for comparison with model predictions at the clinically applied fraction size of 1.8 Gy. As shown in Fig. 4 and Table E2, the predictions of the 3 phenomenological (CRB, WDB, and MNM) and the 3 mechanistic (mMKM, UNIVERSE, and LEM IV) models showed a good agreement when considering the size of the SE. Interestingly, and in contrast to the direct comparison with the experimental data in Fig. 3, LEMIV agrees slightly less with the extrapolated data compared with mMKM and UNIVERSE. Also, the 2 patient-derived models (Bahn et al^25^ and Eulitz et al^26^) performed quite well and have the advantage of being directly derived from patients, although they are only valid for the fraction doses applied to the underlying patient population. In general, the deviation between model prediction and experimental data may originate from the details in the model itself, the selected model parameters, and the way the model is transferred from the in vitro to the in vivo setting. To identify the reasons and improve the model predictions, detailed investigations and additional simulations are required for each model, which were beyond the scope of this study. However, this can be done in further studies.

Whether a variable RBE model will be implemented for patient treatments in the future is a clinical decision that will be guided by the available data on normal tissue toxicity. Several task groups discussed this issue and published consensus reports.^23^^,^^57^, ^58^, ^59^ While the task groups acknowledged an increased end-of-range RBE for protons, especially in normal tissues with a small α/β, it was recommended to consider the associated risk by LET-based treatment planning or by using adapted dose-volume constraints rather than by implementing a full variable RBE model until more clinical evidence on adverse RBE effects and their dependence on treatment planning and patient-specific parameters is available. With respect to this, it was also pointed out that more systematic experimental studies in normal tissues are required to quantify the proton RBE as a function of LET and dose for clinically relevant endpoints.^23^^,^^59^ Following this recommendation, this study established RBE values as a function of LET and dose for the late-reacting tissue of the CNS using a clinically highly relevant endpoint.

## Conclusions

In conclusion, this study provides RBE values of protons in the rat spinal cord as a function of LET and dose. The results clearly demonstrate LET- and dose-dependent RBE, and extrapolation down to 1.8 Gy provides estimates of the RBE at clinically applied fraction sizes. Comparison with several variable RBE models provided relevant information on the potential future implementation of a variable RBE model in clinical practice.

## Disclosures

Christian P. Karger received funding for the present study from the German Cancer Aid (grant numbers 111434 and 70112975) and support for educational lectures and textbook contributions. Michael Scholz holds a European Patent 10 718 875.7 with royalties paid by Siemens and RaySearch. All other authors do not report any financial interests.

## Appendix Supplementary materials

Supplementary material - new Image, application 1

## Footnotes

Supplementary material associated with this article can be found in the online version at doi:10.1016/j.adro.2025.101809.
