# Supplementary material for: Variable Relative Biological Effectiveness of Protons in the Rat Spinal Cord: Measurements and Comparison With Model Calculations
Source: Adv Radiat Oncol. 2025 May 16;10(8):101809. doi: 10.1016/j.adro.2025.101809 (PMC12269397; doi:10.1016/j.adro.2025.101809)
Supplement: Supplementary material - new [file mmc1.pdf]

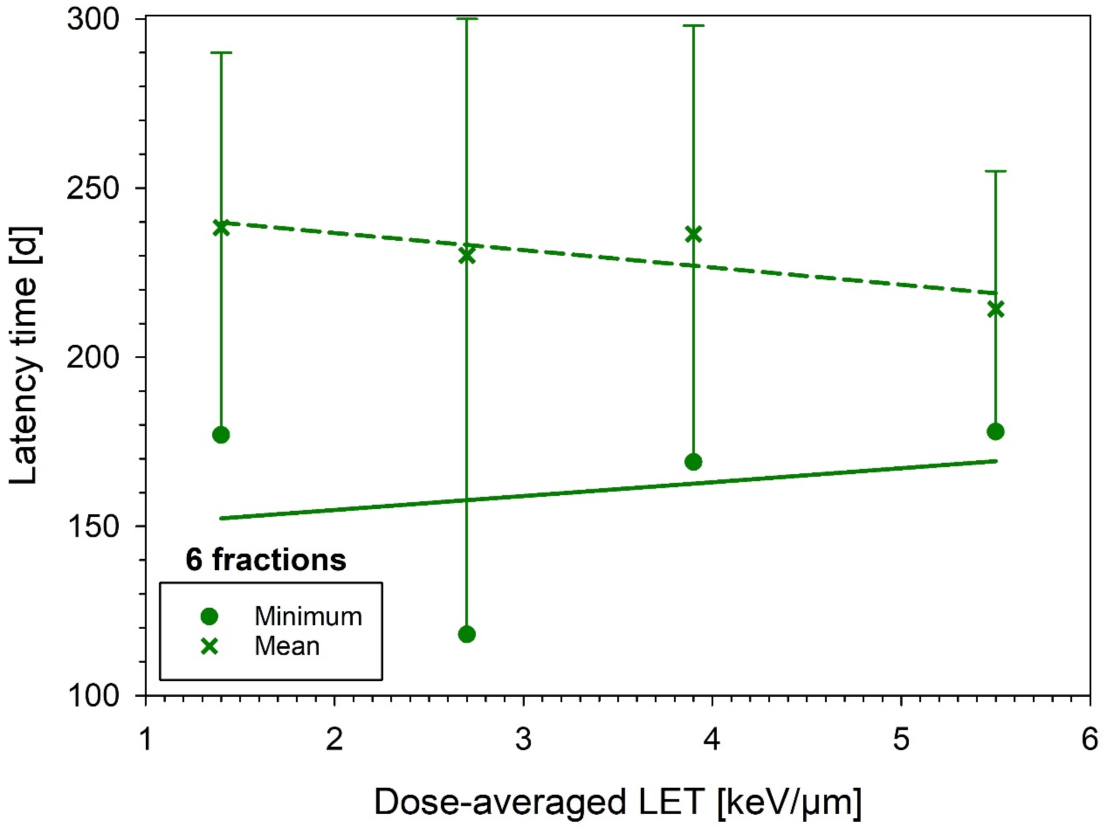


**Figure E1** Minimum and mean latency time until onset of the endpoint paresis grade II after 6 fractions of protons as a function of LET. Error bars indicate the range of latency times.

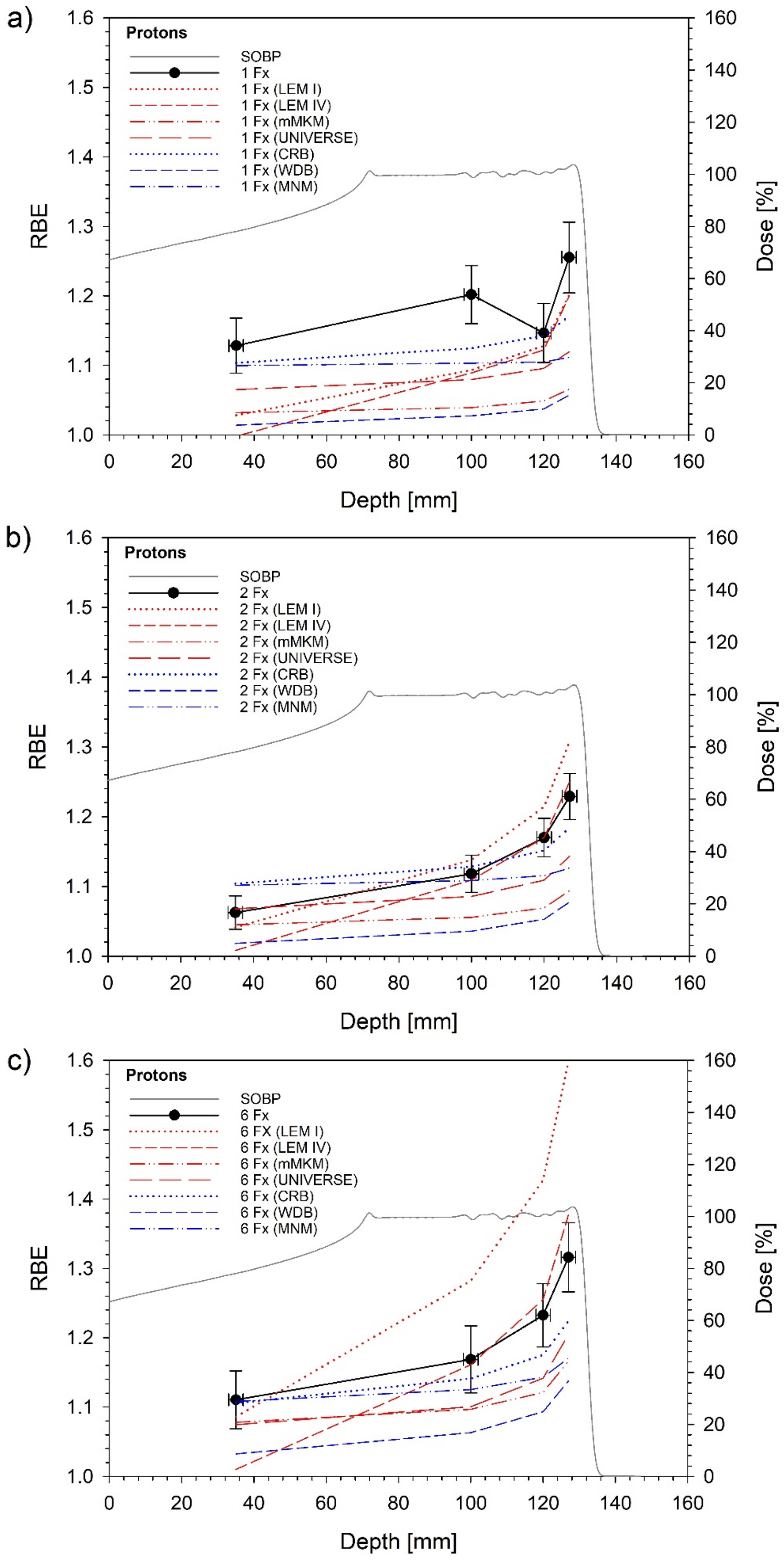


**Figure E2** Comparison of model-predicted and measured relative biological effectiveness (RBE) of protons as a function of depth for single (a), split (b), and six (c) fractions (Fx). Four mechanistic (LEM I, LEM IV, mMKM, UNIVERSE), and three phenomenological (CRB, WDB, MNM) models were used. Experimental data are the same as in Fig. 2c. In addition, the SOBP is visualized.

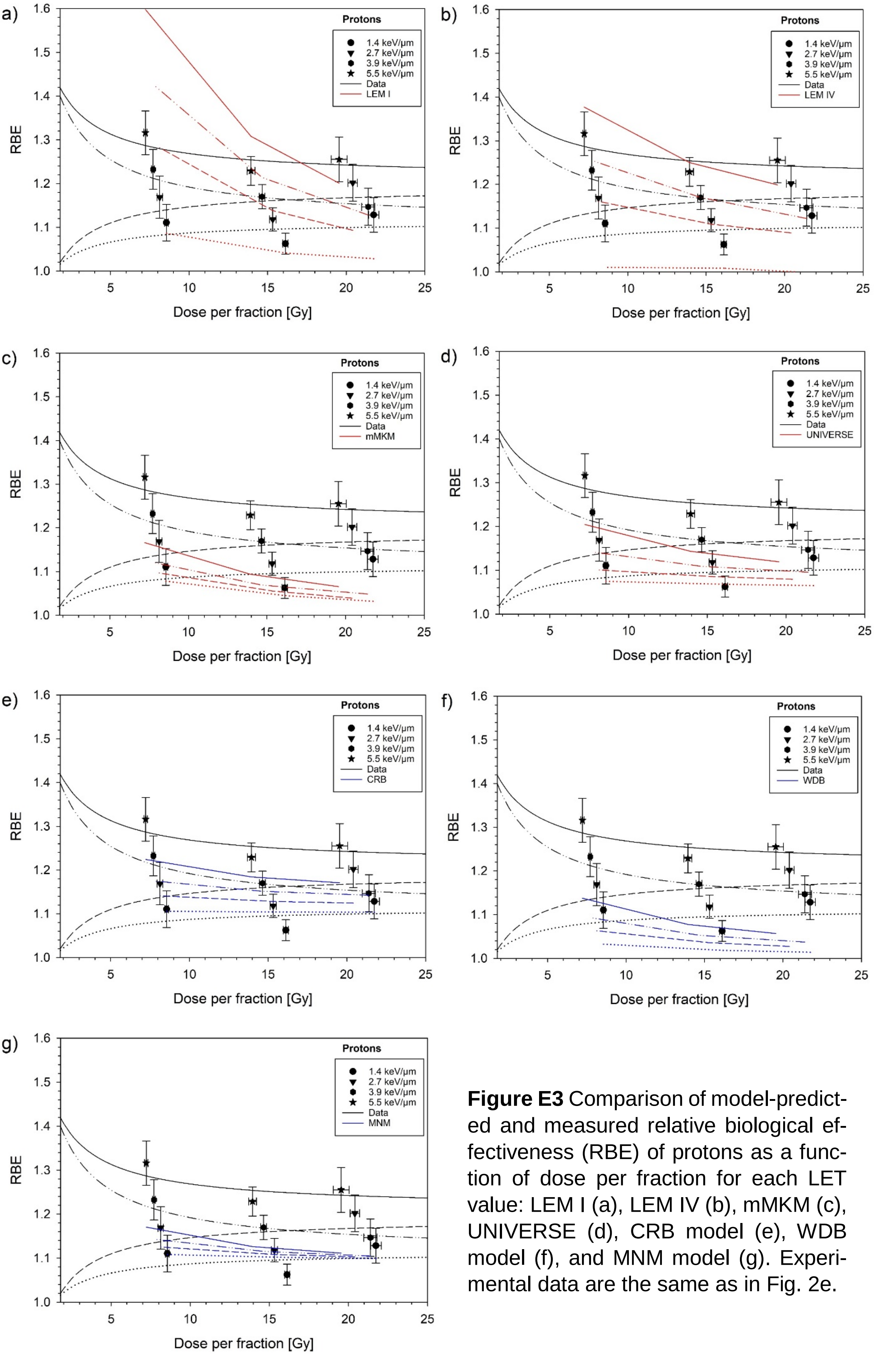


**Figure E3** Comparison of model-predicted and measured relative biological effectiveness (RBE) of protons as a function of dose per fraction for each LET value: LEM I (a), LEM IV (b), mMKM (c), UNIVERSE (d), CRB model (e), WDB model (f), and MNM model (g). Experimental data are the same as in Fig. 2e.

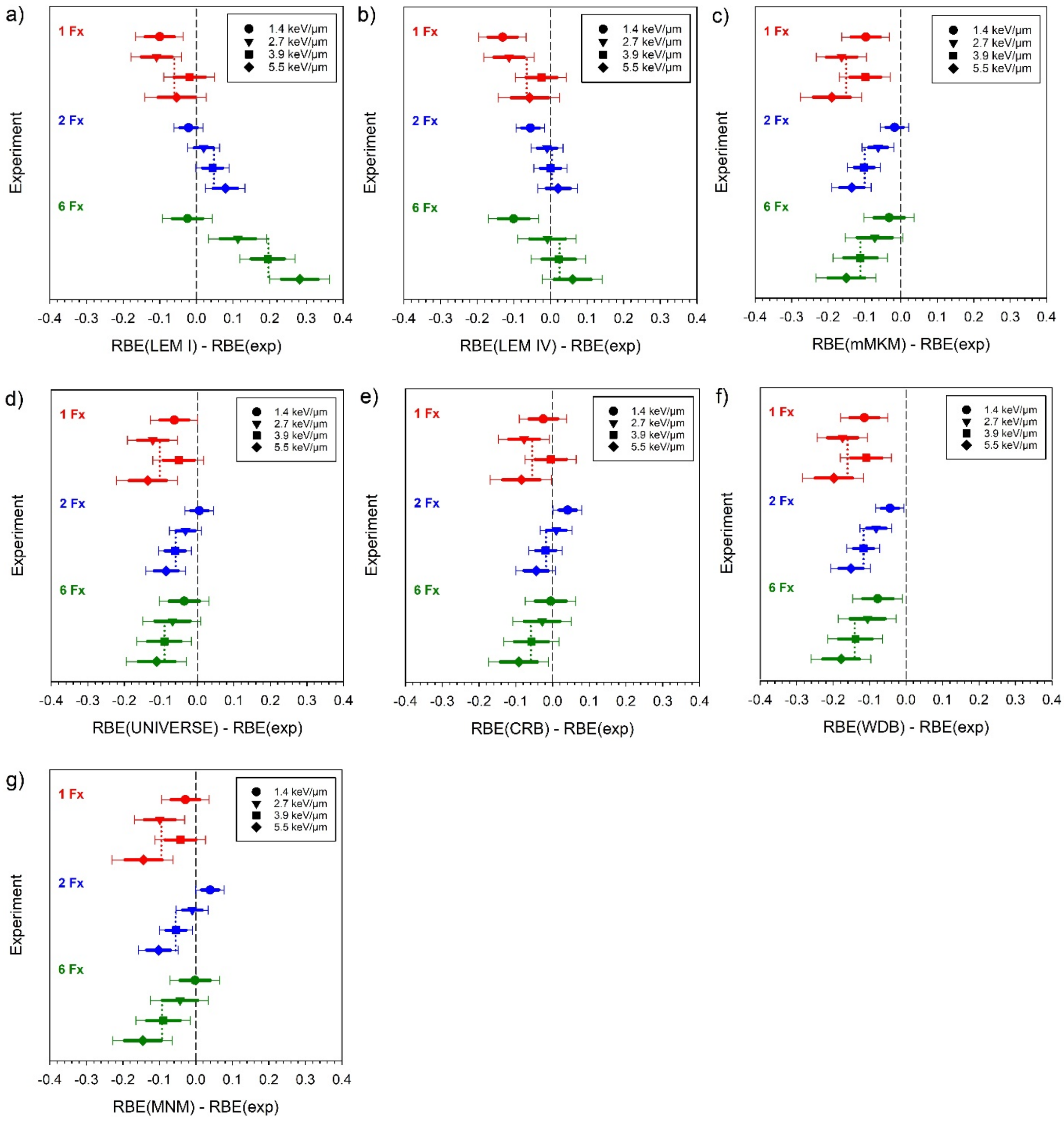


**Figure E4** Deviations between predicted and measured relative biological effectiveness (RBE) values of protons for 1, 2 and 6 fractions and the four different LET values. The graphs display the deviations for the mechanistic LEM I (a), LEM IV (b), mMKM (c), UNIVERSE (d), and the phenomenological CRB (e), WDB (f), and MNM (g) models. Horizontal error bars represent the experimental standard errors (thick line) and the 90% confidence intervals (thin line) provided in Table 2. The vertical dotted lines indicate the average deviation within the three positions of the SOBP for each fractionation schedule. Average deviations and SD over all fractionations are given in table E1, separately for the SOBP and plateau region.

**Table E1 Mean deviation (dev) between predicted and experimental RBE and the corresponding standard deviation (SD), calculated over 1, 2 and 6 fractions. The values are displayed separately for the SOBP and the plateau region. The SD is considered as a uniformity measure of the deviations. In addition, the ranks of the absolute values of both quantities are given.**

| 1, 2 and 6 Fx | Mean dev | Rank | SD | Rank | Mean dev | Rank | SD | Rank |
|---|---|---|---|---|---|---|---|---|
| | SOBP | | | | Plateau | | | |
| LEM I | 0.061 | 3 | 0.123 | 7 | -0.049 | 4 | 0.045 | 7 |
| LEM IV | -0.012 | 1 | 0.050 | 6 | -0.095 | 7 | 0.038 | 5 |
| mMKM | -0.120 | 6 | 0.042 | 4 | -0.049 | 5 | 0.042 | 6 |
| UNIVERSE | -0.084 | 5 | 0.034 | 1 | -0.031 | 3 | 0.035 | 3 |
| Carabe | -0.044 | 2 | 0.037 | 2 | 0.004 | 2 | 0.034 | 1 |
| Wedenberg | -0.140 | 7 | 0.039 | 3 | -0.079 | 6 | 0.035 | 4 |
| McNamara | -0.081 | 4 | 0.047 | 5 | 0.003 | 1 | 0.034 | 2 |

**Table E2 Mean deviation (dev) between predicted and extrapolated experimental RBE and the corresponding standard deviation (SD) at 1.8 Gy per fraction. The values are displayed separately for the SOBP and the plateau region. The SD is considered as a uniformity measure of the deviations. In addition, the ranks of the absolute values of both quantities are given.**

| 1.8 Gy/Fx | Mean dev | Rank | SD | Rank | Dev | Rank |
|---|---|---|---|---|---|---|
| | SOBP | | | | Plateau | |
| LEM I | 1.005 | 9 | 0.172 | 9 | 0.304 | 9 |
| LEM IV | 0.301 | 8 | 0.115 | 1 | 0.004 | 1 |
| mMKM | 0.062 | 5 | 0.165 | 7 | 0.204 | 8 |
| UNIVERSE | 0.010 | 3 | 0.134 | 4 | 0.090 | 2 |
| Carabe | 0.017 | 4 | 0.153 | 5 | 0.099 | 4 |
| Wedenberg | 0.005 | 2 | 0.154 | 6 | 0.091 | 3 |
| McNamara | 0.000 | 1 | 0.168 | 8 | 0.128 | 5 |
| Bahn et al. | 0.126 | 6 | 0.130 | 3 | 0.128 | 6 |
| Eulitz el al. | 0.207 | 7 | 0.123 | 2 | 0.157 | 7 |
